# Attributable Risk and Economic Cost of Cardiovascular Hospital Admissions Due to Ambient Particulate Matter in Wuhan, China

**DOI:** 10.3390/ijerph17155453

**Published:** 2020-07-29

**Authors:** Xuyan Wang, Chuanhua Yu, Yunquan Zhang, Fang Shi, Runtang Meng, Yong Yu

**Affiliations:** 1 Department of Epidemiology and Biostatistics, School of Health Sciences, Wuhan University, Wuhan 430071, China; WangxYan@whu.edu.cn (X.W.); 18204313963@163.com (F.S.); 2 Global Health Institute, Wuhan University, Wuhan 430072, China; 3 Department of Epidemiology and Biostatistics, School of Public Health, Medical College, Wuhan University of Science and Technology, Wuhan 430065, China; YunquanZhang@wust.edu.cn; 4 Hubei Province Key Laboratory of Occupational Hazard Identification and Control, Wuhan University of Science and Technology, Wuhan 430065, China; 5 Department of Preventive Medicine, School of Medicine, Hangzhou Normal University, Hangzhou 311121, China; mengruntang@whu.edu.cn; 6 School of Public Health and Management, Hubei University of Medicine, Shiyan 442000, China

**Keywords:** ambient particulate matter, cardiovascular disease, hospitalizations, attributable hospitalization costs

## Abstract

Although the adverse effects of ambient particulate matter (PM) on cardiovascular disease (CVD) have been previously documented, information about their economic consequence was insufficient. This study aimed to evaluate the attributable risk and economic cost of cardiovascular hospitalizations due to ambient PM. Data of CVD hospitalizations and PM concentrations from 1 January 2015 to 31 December 2017 were collected in Wuhan, China. A generalized additive model was applied to quantify the PM-attributable CVD hospitalizations, and total attributable hospitalization costs were calculated via multiplying the total attributable cases by the case-average hospitalization costs. A total of 45,714 CVD hospitalizations were included in this study. The results showed that a 10 µg/m^3^ increase in PM_2.5_ and PM_10_ concentrations at lag7 day, respectively, contributed to a 1.01% (95% confidence interval: 0.67–1.34) and 0.48% (0.26–0.70) increase in CVD hospitalizations. During the study period, 1487 and 983 CVD hospitalizations were attributable to PM_2.5_ and PM_10_, equaling an economic cost of 29.27 and 19.34 million RMB (1 RMB = 0.1424 USD), respectively, and significant differences in PM-attributable hospitalizations and economic burden were found between gender and age groups. Our study added evidence in heavily polluted megacities regarding the increased health risk and economic cost of CVD hospitalizations associated with ambient particulate pollution.

## 1. Introduction

Ambient particulate matter (PM) is typically considered to be the primary air pollutant, which has been of increasing public concern, and was estimated to have caused 40.93 million deaths and 105.67 million disability-adjusted life-years (DALYs) in 2016 globally [1]. Particularly, ambient PM contributed greater health impacts to Chinese populations [2]. Emerging studies have evaluated the adverse effects of PM on cardiovascular disease (CVD). For instance, with a 10 µg/m^3^ increase of the PM_2.5_ (particulate matter with aerodynamic diameter <2.5 µm) concentration, a nationwide time-series analysis in 272 Chinese cities suggested that PM_2.5_ was related to an increase in daily cardiovascular disease mortality of 0.27% (95% confidence interval: 0.18–0.36) [3]; other studies [4,5] found that exposure to PM_2.5_ contributed to increased cardiovascular hospital admissions in Shandong (0.40% (0.03–0.78)), and Beijing (0.30% (0.20–0.39)) as well. For PM_10_ (particulate matter with aerodynamic diameter <10 µm), a review study reported a 0.70% (0.60–0.80) increase in cardiovascular mortality per 10 µg/m^3^ increase of the PM_10_ concentration [6]; PM_10_ exposure was also associated with increased ischemic heart disease mortality (0.86% (0.22–1.51)) [7] and elevated stroke hospitalizations (1.0% (0.1–1.4)) [8].

The health costs of air pollution were predicted to achieve $580 billion globally by 2050 [9]. Meanwhile, a study indicated that PM_2.5_ pollution should cause a loss of about 2.0% of China’s GDP by 2030 if necessary measures are not taken [10]. There were 290 million CVD cases in China [11], and CVD accounted for the highest proportion of the total curative care expenditure followed by neoplasm and respiratory diseases [12]. Nevertheless, few studies have determined the economic burdens of CVD attributable to ambient PM, and previous studies have mainly focused on its health outcomes (e.g., mortality and hospital admission), as described above. Therefore, the assessment of both the health and economic burden is urgently needed, which could provide more comprehensive information for the cost-effectiveness evaluation of policy-making regarding air pollution control.

It was on this basis we conducted our study to determine the association between exposure to ambient PM (including PM_2.5_ and PM_10_) and cardiovascular hospitalizations in Wuhan, China. In addition, we further quantified the CVD hospitalizations and hospitalization expenses attributable to PM_2.5_ and PM_10_; the preventable CVD hospitalizations and savable hospitalization costs were also estimated based on the scenarios that the concentrations of PM_2.5_ and PM_10_ during the study period could be maintained at relatively lower levels.

## 2. Methods

### 2.1. Study Area

As the largest metropolis in central China, Wuhan has experienced heavy air pollution in the past few years with the development of urbanization, motorization and industrialization [13]. Wuhan is located at 113.7–113.1° E and 29.9–31.4° N with an 8569.15 km^2^ total metropolitan area, and the permanent residents were 10.33 million by the end of 2019. The main climate type in Wuhan is humid subtropical monsoon, characterized by hot summers and cold winters as well as short springs and autumns.

### 2.2. Data Collection

Daily hospitalization admission data between 1 January 2015 and 31 December 2017 were obtained from two general hospitals in Wuhan; both the chosen hospitals are Grade-A tertiary hospitals with well-known expertise in diagnosing and treating disease. Records of hospital admissions included admission date, age, gender, principal discharge diagnosis and hospitalization costs, and the diagnosis of disease was coded according to the International Classification of Disease Tenth Revision (ICD-10). Patients hospitalized due to CVD (ICD-10: I00-I99) were included in our study; the entire study group was divided into two gender groups (male and female) and two age groups (0–64 years and +65 years). Further break-down of the 0–64 years group was not conducted because the children group (0–14 years) only accounted for 0.24% of the total cases. Besides, two subtypes of CVD including ischemic heart disease (IHD, ICD-10: I20-I25) and stroke (ICD-10: I60-I69) were also analyzed.

Data of ambient air pollutants including PM_2.5_, PM_10_, SO_2_ (sulfur dioxide) and NO_2_ (nitrogen dioxide) were acquired from the Hubei Environmental Protection Bureau; the daily mean concentration (µg/m^3^) for each air pollutant was calculated by averaging across the measurements from the 10 monitoring stations in Wuhan. Daily meteorological data were collected from the China Meteorological Data Network (http://data.cma.cn), including mean air temperature (°C) and relative humidity (%). The spatial distribution of included hospitals and air monitoring sites are displayed in Figure S1.

### 2.3. Data Analysis

A three-stage analytic approach was used to analyze the data. Firstly, we decomposed the time-series data (including PM concentration, CVD hospitalizations and hospitalization costs) to detect a potential long-term trend and seasonality. Secondly, we applied a time-series model to estimate PM-hospitalization associations. Thirdly, we calculated the hospitalizations and hospitalization costs attributable to PM_2.5_ and PM_10_.

#### 2.3.1. First-Stage Analysis

A seasonal-trend decomposed method was used to detect a potential long-term trend and seasonality of the daily concentration of PM (PM_2.5_ and PM_10_), daily hospitalizations and case-average hospitalization costs. The time-series was split into three additive components, including a long-term trend during the study period, seasonal variations within years and random variation [14,15]; the fundamental statistical model was as shown:

(1) $$Y_{t}=T_{t}+S_{t}+R_{t}$$

where $T_{t}\;$ is the linear trend; $S_{t}$ is the seasonal effect; $I_{t}\;$ is the r random noise and *t* = 1, 2, …., N.

#### 2.3.2. Second-Stage Analysis

The associations of PM_2.5_ and PM_10_ with CVD hospitalizations were estimated by using a generalized additive model (GAM) with quasi-Poisson regression as shown:

(2) $$\log\left(E_{i}\right)=\beta_{i}\left(PMC_{i}\right)+s\left(TIME,df\right)+s\left(MT,df\right)+s\left(RH,df\right)+DOW+a$$

where $E_{i}$ is the expected hospitalizations on day *i*, $\beta_{i}\;$ is the estimated slope of associations of the concentrations of PM_2.5_ or PM_10_ on day *i*, *s* is a spline smoothing function for the nonlinear variable (e.g., time, mean temperature and relative humidity), *df* is the degree of freedom—7 *df* per year for time trends [15,16] and 3 *df* per year for mean temperature and relative humidity were defined [17], *DOW* is a categorical variable for day of the week and α is the intercept of the model.

Effect estimates across lag0 day (the day of hospital admission) to lag7 day (7 days prior to hospital admission) were investigated in our current study, which were presented as a percent change (*PC*, %), and the 95% confidence interval (CI) in daily CVD hospitalizations was associated with a 10 µg/m^3^ increase in the concentrations of PM_2.5_ or PM_10_. *PC* was calculated through Equation (3):

(3) $$PC=\left[\exp\left(\beta_{i}\times 10\right)-1\right]\times 100$$

where $\beta_{i}\;$ is the estimated slope of associations of the concentrations of PM_2.5_ or PM_10_ on day *i*, which was obtained from Formula (2).

#### 2.3.3. Third-Stage Analysis

The attributable fraction (*AF*) and attributable number (*AN*) of CVD hospitalizations due to PM_2.5_ or PM_10_ were estimated by using the method mentioned previously [18,19,20,21]. The following, Equations (4) and (5), were used:

(4) $$AF_{i}=1-\frac{1}{\exp\left(\beta_{i}*\Delta C_{i}\right)}$$

(5) $$AN_{i}=AF_{i}\times N_{i}$$

where $\;AF_{i}\;$ is the attributable fraction on day *i,* $AN_{i}\;$ is the attributable number of CVD hospitalizations on day *i*, $N_{i}\;$ is the daily CVD hospitalizations on day *i* and $\beta_{i}\;$ is the estimated slope of associations of the concentrations of PM_2.5_ or PM_10_ on day *i*. $\Delta C_{i}$ is the concentration difference between the observed and the reference concentrations of PM_2.5_ or PM_10_ on day *i*. The reference concentration is a threshold level at which no health effects are yet assumed, then the air quality standard proposed by the World Health Organization (WHO) was considered as the threshold (24 h average value: 25 µg/m^3^ for PM_2.5_ and 50 µg/m^3^ for PM_10_) in our current study [20].

Therefore, the total attributable number was estimated by summing daily AN, and its ratio with total CVD hospitalizations was the total *AF*. We further calculated the total attributable hospitalization costs via multiplying the total attributable number by the case-average hospitalization costs during the study period [20,22]. The potential number of avoidable hospitalizations and savable hospitalization costs were also estimated based on the scenarios that the concentrations of PM_2.5_ and PM_10_ during the study period could be maintained at relatively lower levels. All the costs were presented as the 2020 price in Renminbi using a consumer price index for adjustment (1 RMB = 0.1424 USD).

#### 2.3.4. Sensitivity Analysis

A sensitivity analysis was performed to examine the robustness of the model through: (1) adjusting for co-pollutants (SO_2_ and NO_2_) and (2) changing the degree of freedom (*df* = 8 and 9) for the long-term trend and seasonality.

All analyses were conducted by R software (version 3.6.1, R Foundation for Statistical Computing, Vienna, Austria), and two-sided tests with *p* < 0.05 were considered to be statistically significant.

### 2.4. Ethical Approval

All the data in our study were anonymous and de-identified, and it was approved by the Ethics Committee of the Medical Department of Wuhan University to waive informed consent of the participants (No. 2019YF2037).

## 3. Results

A total of 45,714 CVD hospitalizations from 1 January 2015 to 31 December 2017 were included in this study, of which IHD and stroke, respectively, accounted for 20.77% and 38.29%. Table 1 provides the summary statistics of daily hospitalization counts, hospitalization costs, air pollutant concentration and meteorological factors in Wuhan, China, from 2015–2017. On average, there were 42 admission cases per day for CVD, 9 cases for IHD and 16 cases for stroke. The daily CVD hospitalizations of males were obviously higher than that of females, and there were less daily CVD hospitalizations for patients aged 0–64 years compared with the older group (65+ years). The average hospitalization costs for CVD, IHD and stroke were 19,680 RMB, 19,320 RMB and 24,290 RMB, respectively. Higher mean CVD-associated costs were observed for males and patients aged 0–64 years.

The annual average concentrations of PM_2.5_ and PM_10_ were 58.52 µg/m^3^ and 96.10 µg/m^3^ in Wuhan; both exceeded the secondary standard of ambient air quality in China (annual average value: 35µg/m^3^ for PM_2.5_ and 70 µg/m^3^ for PM_10_). In addition, the annual mean temperature and relative humidity were 17.20 °C and 80.62%, respectively. Spearman’s rank correlation indicated that PM_2.5_ and PM_10_ were moderately correlated with SO_2_ and NO_2_, while they were slightly correlated with meteorological factors (see Table S1).

**Figure 1:**
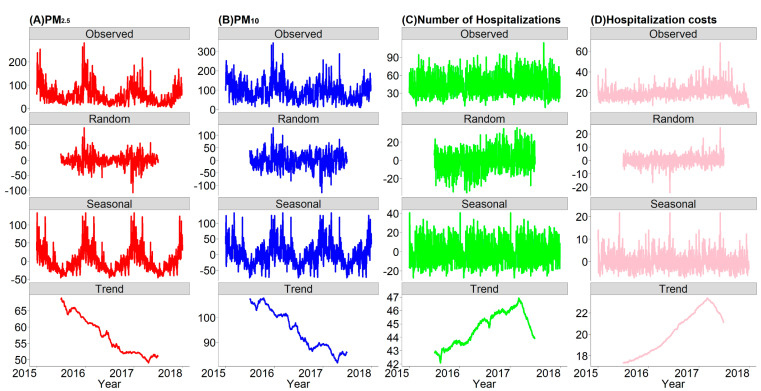
The decomposed distributions for the daily concentrations of PM_2.5_ and PM_10_, daily hospitalizations and case-average hospitalization costs due to CVD in Wuhan, China, from 2015–2017. (**A**) daily concentration of PM_2.5_; (**B**) daily concentration of PM_10_; (**C**) daily number of hospitalizations; (**D**) case-average hospitalization costs.

The long-term trend and seasonality of the daily concentrations of PM_2.5_ and PM_10_, daily hospitalizations and case-average hospitalization costs due to CVD are shown in Figure 1. During the study period, the daily concentrations of PM_2.5_ and PM_10_ had similar characteristics of periodic fluctuation with a downtrend annually, the seasonal pattern of which showed a high concentration in winter and spring while a low concentration in summer and autumn. However, daily CVD hospitalizations and case-average hospitalization costs increased over the three-year study period without notable seasonal fluctuations. Similar trends in hospitalizations and costs were also noted for IHD and stroke (see Figure S2).

**Figure 2:**
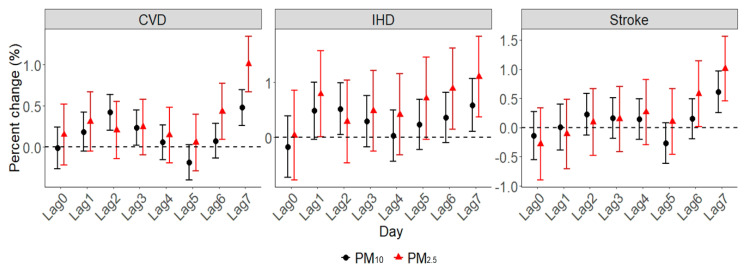
The estimated percent change of cardiovascular disease (CVD) hospitalizations due to a 10 µg/m^3^ increase in PM_2.5_ and PM_10_ concentrations.

Figure 2 illustrates the estimated percent changes (%, 95% CI) of hospitalization risks associated with a 10 µg/m^3^ increase in PM_2.5_ and PM_10_ concentrations at different lag days in single-pollutant models. We found evidence for significant positive associations for at least two exposure lag days between the PM_2.5_ concentration and hospital admissions for all cardiovascular outcomes. The strongest effects were noted at lag7 day; the corresponding percent changes of hospitalizations for CVD, IHD and stroke were 1.01% (0.67–1.34), 1.10% (0.37–1.84) and 1.01% (0.45–1.56), respectively. With a 10 µg/m^3^ increase in PM_10_, significant percent changes of cardiovascular hospitalizations first occurred at lag2 day with the exception of strokes. Likewise, estimated values reached the peak at lag7 day, and were 0.48% (0.26–0.70), 0.58% (0.11–1.07) and 0.61% (0.25–0.96) for CVD, IHD and stroke, respectively. To ease the interpretation, the lag7 day concentrations of PM_2.5_ and PM_10_ are further analyzed in the following paragraphs, since these days produced the largest effect estimates.

**Figure 3:**
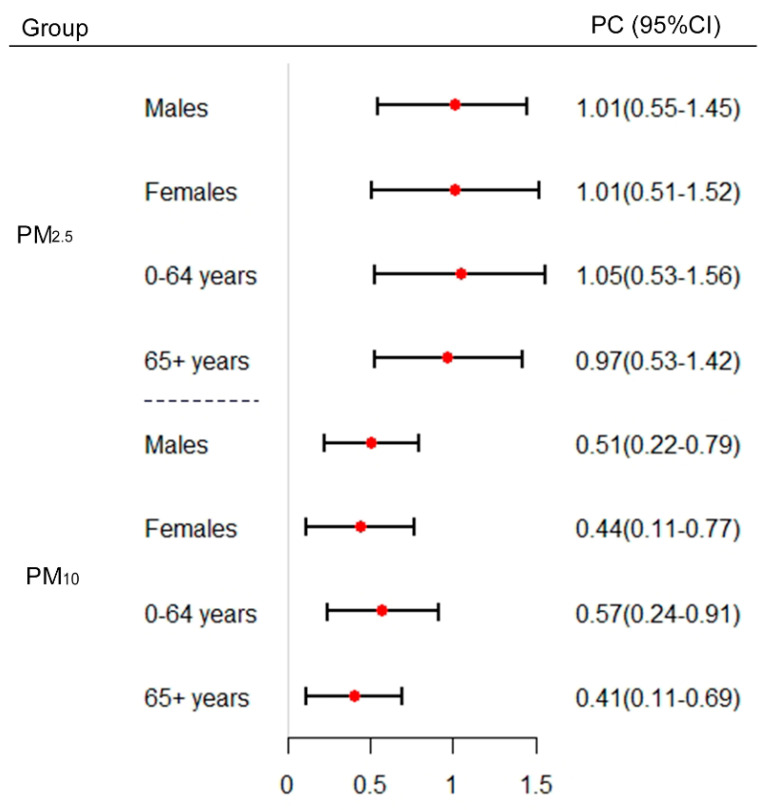
The estimated percent change of CVD hospitalizations per 10 µg/m^3^ increase in PM_2.5_ and PM_10_ concentrations, by gender and age group. PC: percent change; CI: confidence interval.

Results of subgroup analyses of CVD hospitalizations are presented in Figure 3. There was no notable gender difference for PM_2.5_, whereas males were more vulnerable to PM_10_ (0.51% (0.22–0.79)) than females (0.44% (0.11–0.77)). For age group, greater estimated values were found for the group aged 0–64 years; the percent changes of PM_2.5_ and PM_10_ were 1.05% (0.53–1.56) and 0.57% (0.24–0.91), respectively. Subgroup analyses of IHD and stroke are presented in Figures S3 and S4, and significant differences were found by gender and age groups.

Table 2 demonstrates the estimated attributable figures for different CVD observations during the study period. For all CVD, 1487 and 983 hospitalizations were, respectively, attributable to PM_2.5_ and PM_10_, which corresponded to 29.27 million RMB and 19.34 million RMB. Both attributable hospitalizations and attributable hospitalization costs were higher in males than in females. In addition, there were more attributable hospitalizations due to PM_2.5_ for the group aged 65+ years than the younger group (0–64 years), while the opposite results were produced by PM_10_. In addition, there were 340 and 249 IHD-related hospitalizations attributable to PM_2.5_ and PM_10_, and the associated attributable hospitalization costs were 6.57 million RMB and 4.82 million RMB, respectively. For stroke, 13.35 million RMB and 8.98 million RMB were attributable to PM_2.5_ and PM_10_, and the attributable hospitalizations were 550 and 465, respectively.

**Figure 4:**
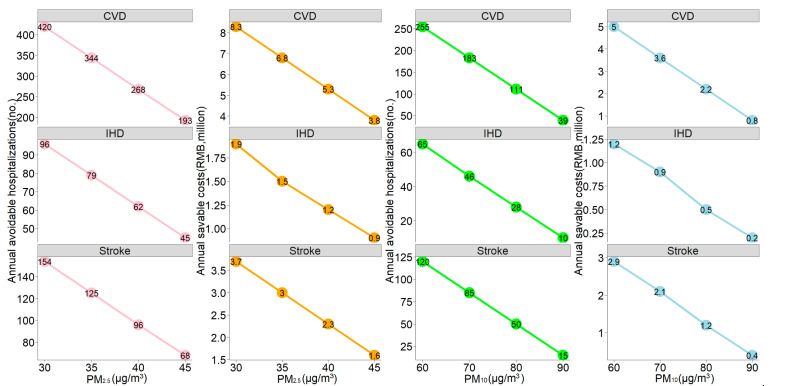
The estimated annual avoidable hospitalizations and savable hospitalization costs if the historical concentrations of PM_2.5_ and PM_10_ could be maintained at relatively low levels. The air quality standard proposed by the WHO (24-h average value: 25 µg/m^3^ for PM_2.5_ and 50 µg/m^3^ for PM_10_) was considered as the reference.

Figure 4 illustrates the potential number of avoidable hospitalizations and savable hospitalization costs if the concentration of PM_2.5_ and PM_10_ during the study period could be maintained at relatively low levels. For total CVD, maintaining the PM_2.5_ concentration at 45 µg/m^3^ could prevent 193 hospitalizations and save 3.8 million RMB annually. In addition, if the PM_10_ concentration could be maintained at 90 µg/m^3^, 39 hospitalizations could be avoided annually, reducing 0.8 million RMB correspondingly. It seems obvious that more hospital admissions and hospitalization costs could be avoided if the historical concentrations of PM_2.5_ and PM_10_ are kept at lower levels, such that if the PM_2.5_ concentration is kept at 30 µg/m^3^, 420 hospitalizations and 8.3 million RMB could be avoided annually, and if the concentration of PM_10_ is maintained at 60 µg/m^3^, 255 hospitalizations and 5.0 million RMB could be averted annually. In addition, more health and economic benefits could be obtained for stroke than IHD. For instance, if the historical concentration of PM_2.5_ is maintained at 45 µg/m^3^, more stroke-associated hospitalizations and costs (68 and 1.5 million RMB, respectively) could be prevented than IHD (45 and 0.9 million RMB, respectively). More details about the subgroup analysis are showed in the supplementary material (see Figure S5).

Sensitivity analyses of adjusted co-pollutants in the model (SO_2_ and NO_2_) and changed *df* (8 and 9) for the long-term trend and seasonality were performed, which suggested that our main models were generally robust (See Table S2).

## 4. Discussion

Some notable results were found in this time-series study in Wuhan, China. First, the daily concentration of ambient PM showed a downtrend, while daily cardiovascular hospitalizations and hospitalization costs increased during the study period. Second, exposure to PM_2.5_ and PM_10_ were associated with an excess risk of CVD hospitalization, and differences were found by gender and age groups. Third, substantial economic costs were attributed to PM_2.5_ and PM_10_ exposure; millions of hospitalization costs could be avoided if the historical PM concentration is maintained at relatively low levels.

In this study, we conducted a decomposed method to detect the potential long-term trend and seasonality of the daily concentration of ambient PM, daily hospitalizations and case-average hospitalization costs. Possibly as a result of strengthened measures taken by the local government to control ambient air pollution recently, a trend of a decline in the PM concentration from 2015 to 2017 was observed. The ambient PM concentration was relatively high in winter and spring; such a seasonal pattern could be interpreted by the increased burning of coal to provide central heating in the cold season that might cause more PM. Another reason might be that temperature inversion is common in the cold season, which would inhibit the spread of air pollutants. However, both daily CVD hospitalizations and corresponding average hospitalization costs performed uptrends during the study period, echoing a study [23] that suggested that the prevalence of non-communicable diseases was increasing worldwide, especially for CVD. A natural cubic spline for time with 7 *df* per year was commonly used to control for the long-term trend of daily hospital admissions [15,16,24]. In addition, with the growth of per-capita income and chronic disease morbidity, rates of patients leaving against medical advice and avoiding the hospital decreased [25], contributing to the increase of hospitalization expenses.

In line with previous studies, our results reported that PM was associated with an excess risk of CVD hospitalization. For example, with a 10 µg/m^3^ increment of the PM concentration, a 0.87% increase in cardiovascular hospital admissions due to PM_2.5_ [13] and 1.0% increase in stroke hospitalizations due to PM_10_ [8] were found in Wuhan. In addition, other researchers observed consistently increased hospitalizations associated with PM_2.5_ and PM_10_ for CVD in some Chinese regions such as Shandong [4], Beijing [5], Shanghai [26] and Sichuan [27]. Moreover, studies [28,29] of Brazil, France, Iran and Italy also demonstrated the positive associations between PM exposure and cardiovascular hospitalizations. Ambient PM mainly comes from fossil fuel combustion and automobile exhaust, which contains toxic organic compounds such as benzopyrene and many heavy metals (e.g., lead, nickel and chromium) [30]. Previous clinical and toxicological studies have established several possible mechanisms for how PM and its components adversely impact the cardiovascular system. For instance, Cao et al. [31] indicated that PM could lead to cardiomyocyte apoptosis by protein kinase activation, and Kowalska et al. [32] suggested that PM contributed to myocardial infarction via destabilizing atherosclerotic plaques. Other researchers also found that PM was related to acute decompensated heart failure symptoms [33,34], decreased heart rate variability and changed autonomic tone [35,36].

Gender and age differences in associations of particulate pollution with cardiovascular health have been of wide interest in air pollution epidemiology. We found that the impacts of PM on CVD were significantly greater in males, which is consistent with a study in Shanghai [26], while other studies [5,37] showed that the gender differences were statistically insignificant. Similar with previous studies [13,38], a greater increase of CVD hospitalizations for the younger group (0–64 years) was also observed in our current study. However, a study of Powell et al. [39] suggested stronger associations between daily CVD hospital admissions and particulate air pollution were observed for the elderly. Considering the gender and age group specific associations were varied, the likely explanation for the heterogeneity might be various study designs, research periods and sociodemographic and economic characteristics.

Economic burden assessment is crucial for the cost-effectiveness evaluation of policy-making regarding air pollution control. A study of Tianjin [40] calculated that attributable CVD mortalities due to PM_2.5_ were equivalent to 2.79% of the local GDP when converted into present value, and Cheng et al. [16] demonstrated that 21 million RMB CVD-associated hospitalization expenses could be saved annually in Lanzhou if the concentration of CO is kept below 1 mg/m^3^. Other studies in regard to mental disorders [20] and pneumonia [22] also showed that a certain amount of medical expenses could be avoided with the decline of PM concentrations. Correspondingly, our current study illustrated that 29.27 million RMB and 19.34 million RMB were, respectively, attributed to PM_2.5_ and PM_10_, and more hospitalization costs could be saved if the historical concentration of PM is maintained at lower levels. To some extent, our study might fill some of the gaps of knowledge about the health economic aspects of particulate air pollution on CVD in central China, and further studies should focus on other air pollutants on cardiovascular health.

The present study had some limitations. First, the average concentration of PM in Wuhan was considered as personal exposure, which may cause measurement errors. It is a well-recognized inherent limitation of such environmental epidemiological studies [41] that is likely to underestimate the effects of PM [42]. Second, the influences of meteorological factors, day of the week, gender and age were considered in our current study, and more individual information such as socio-economic status and behavioral factors could also be considered in follow-up studies. Third, the economic burden calculated in this study tended to understate the real economic burden of CVD due to PM, since the economic cost in our results only included the direct hospitalization expenses, while the indirect medical costs and outpatient expenditures were not analyzed. Consequently, more research should be undertaken to resolve these issues.

## 5. Conclusions

In conclusion, we found that ambient particulate pollution increased CVD risk and cost among hospital admission patients in Wuhan, one of the heavily polluted megacities in central China. Then, we should continue to strengthen the efforts to alleviate the level of particulate pollution in Wuhan, which would certainly reduce the health and economic burden among patients with CVD. Our study also highlights the demand for evaluating the air pollution-related economic effect. It may have important implications for promoting the cost-effectiveness evaluation of measures regarding air pollution control, thereby providing informative priorities about measures with the largest benefits for local decision-makers from the public health perspective.

## Figures and Tables

**Table 1:** Summary statistics of daily hospitalization counts, hospitalization costs, air pollutant concentration and meteorological factors in Wuhan, China, from 2015–2017.

Variable	Mean	SD	Min	Percentile			Max
				P_25_	P_50_	P_75_	
Daily hospitalization counts							
CVD	42	17.79	2	28	41	53	97
IHD	9	4.45	0	5	8	12	25
Stroke	16	6.62	0	11	16	20	43
Males	24	10.76	2	15	23	31	58
Females	18	8.25	0	12	17	23	46
0–64 years	18	8.77	0	12	17	23	53
64+ years	24	10.34	1	16	23	31	60
Hospitalization costs (in 1000 RMB)							
CVD	19.68	31.08	0.04	6.25	10.05	19.12	1036.81
IHD	19.32	25.59	0.13	6.70	10.30	20.70	1036.81
Stroke	24.29	37.32	0.07	6.76	11.78	23.84	946.95
Males	21.22	32.64	0.04	6.53	10.94	21.16	1036.81
Females	17.68	28.79	0.08	5.95	9.01	16.49	728.54
0–64 years	21.22	34.91	0.04	5.84	9.44	18.20	657.65
64+ years	18.53	27.78	0.07	6.58	10.41	19.62	1036.81
Air pollutants (µg/m^3^)							
PM_2.5_	58.52	38.56	4	31	49	76	281
PM_10_	96.10	51.10	10	60	90	124	618
SO_2_	13.39	8.85	3	7	11	17	74
NO_2_	46.17	19.37	11	31	43	57	119
Meteorological factors							
Mean temperature (°C)	17.20	8.94	−3.8	9.2	18	24.7	33.9
Relative humidity (%)	80.62	10.38	42	74	82	88	100

Notes: CVD, cardiovascular disease; IHD, ischemic heart disease; SD, standard deviation; P_25_, 25th percentile; P_50_, 50th percentile; P_75_, 75th percentile.

**Table 2:** The number of hospitalizations and hospitalization costs attributable to PM_2.5_ and PM_10_ in Wuhan, China, from 2015–2017.

Variable	Attributable Hospitalizations (No.)		Attributable Hospitalization Costs (RMB, Million)	
	PM_2.5_	PM_10_	PM_2.5_	PM_10_
CVD	1487 (1007, 1956)	983 (541, 1415)	29.27 (19.81, 38.85)	19.34 (10.65, 27.85)
IHD	340 (119, 549)	249 (49, 441)	6.57 (2.29, 10.53)	4.82 (0.94, 8.53)
Stroke	550 (245, 834)	465 (199, 722)	13.35 (6.16, 20.94)	8.98 (3.84, 13.94)
Males	855 (777, 932)	460 (388, 531)	18.14 (16.48, 19.27)	9.76 (8.24, 11.27)
Females	653 (335, 959)	390 (95, 675)	11.54 (5.91, 16.93)	6.89 (1.68, 11.93)
0–64 years	653 (339, 955)	497 (212, 773)	13.85 (7.19, 20.40)	10.55 (4.51, 16.40)
65+ years	1118 (1035, 1199)	471 (393, 548)	20.71 (19.18, 22.15)	8.72 (7.28, 10.15)

Notes: The air quality standard proposed by the WHO (24-h average value: 25 µg/m^3^ for PM_2.5_ and 50 µg/m^3^ for PM_10_) was considered as the reference.

## Supplementary Materials

The following are available online at https://www.mdpi.com/1660-4601/17/15/5453/s1. Figure S1: The spatial distribution of included hospitals and air monitoring sites in Wuhan, China. Figure S2: The decomposed distribution of daily hospitalizations (no.) and case-average hospitalization costs due to IHD (A and B) and stroke (C and D) in Wuhan, China, from 2015–2017. Figure S3: The estimated percent change of IHD hospitalizations of per 10 µg/m^3^ increase in PM_2.5_ and PM_10_ concentrations, by gender and age group. PC: percent change, CI: confidence interval. Figure S4: The estimated percent change of stroke hospitalizations per 10 µg/m^3^ increase in PM_2.5_ and PM_10_ concentrations, by gender and age group. PC: percent change, CI: confidence interval. Figure S5: The estimated annual avoidable hospitalizations and savable hospitalization costs for CVD subgroups if the historical concentrations of PM_2.5_ and PM_10_ could be maintained at relatively low levels. The air quality standard proposed by the WHO (24-h average value: 25 µg/m^3^ for PM_2.5_ and 50 µg/m^3^ for PM_10_) was considered as the reference. Table S1: The coefficient of the Spearman rank correlation between particulate matter (including PM_2.5_ and PM_10_) and SO_2_, NO_2_ and meteorological factors in Wuhan, China. Table S2: Results of sensitivity analyses by adjusting for co-pollutants and changing the degree of freedom for the long-term trend and seasonality. Results are shown in percent change (%) per 10 µg/m^3^ increase in PM_2.5_ and PM_10_ concentrations at the best lag day.
