# Supplementary material for: Attributable Risk and Economic Cost of Cardiovascular Hospital Admissions Due to Ambient Particulate Matter in Wuhan, China
Source: Int J Environ Res Public Health. 2020 Jul 29;17(15):5453. doi: 10.3390/ijerph17155453 (PMC7432018; doi:10.3390/ijerph17155453)
Supplement: Supplementary file 1 [file ijerph-17-05453-s001.pdf]

Supplementary Materials

Table of contents

Figure S1. The spatial distribution of included hospitals and air monitoring sites in Wuhan, China.

Figure S2. The decomposed distribution of daily hospitalizations (no.) and case-average hospitalization costs due to IHD (A and B) and stroke (C and D) in Wuhan, China, from 2015-2017.

Figure S3. The estimated percent change of IHD hospitalizations per 10 μg/m^3 increase in $PM_{2.5}$ and PM_{10} concentrations, by gender and age group. PC： percent change, CI: confidence interval.

Figure S4. The estimated percent change of stroke hospitalizations per 10 μg/m^3 increase in $PM_{2.5}$ and PM_{10} concentrations, by gender and age group. PC： percent change, CI: confidence interval.

Figure S5. The estimated annual avoidable hospitalizations and savable hospitalization costs for CVD subgroups if the historical concentrations of $PM_{2.5}$ and PM_{10} could be maintained at relatively low levels. The air quality standard proposed by the WHO (24-h average value: 25 μg/m^3 for $PM_{2.5}$ and 50 μg/m^3 for PM_{10}) was considered as the reference.

Table S1. The coefficient of the Spearman rank correlation between particulate matter (including $PM_{2.5}$ and PM_{10}) and SO_2, NO_2 and meteorological factors in Wuhan, China.

Table S2. Results of sensitivity analyses by adjusting for co-pollutants and changing the degree of freedom for the long-term trend and seasonality. Results are shown in percent change (%) per 10 μg/m^3 increase in $PM_{2.5}$ and PM_{10} concentrations at the best lag day.

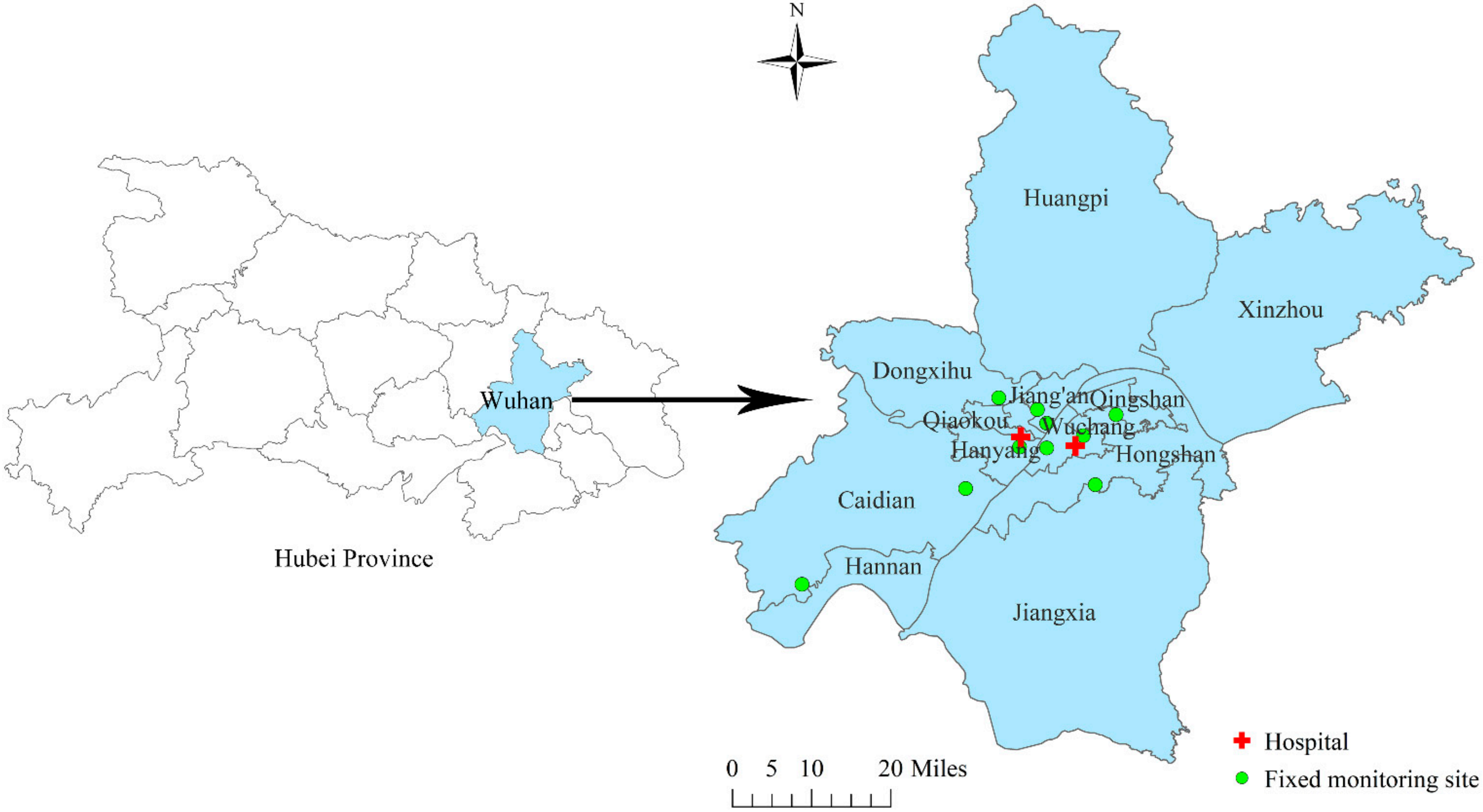

Figure S1. The spatial distribution of included hospitals and air monitoring sites in Wuhan, China.

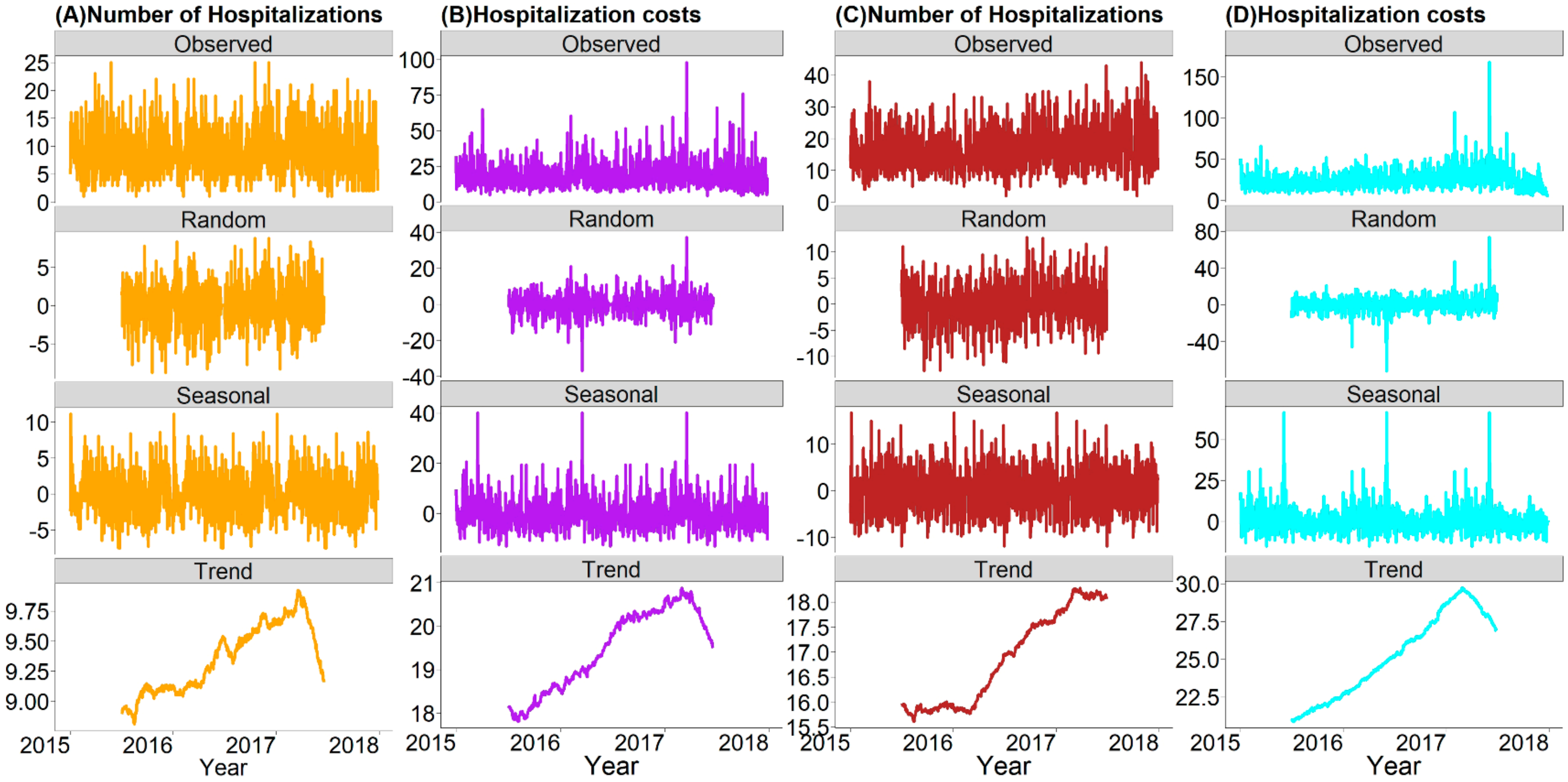

Figure S2. The decomposed distribution of daily hospitalizations (no.) and case-average hospitalization costs due to IHD (A and B) and stroke (C and D) in Wuhan, China, from 2015 –2017.

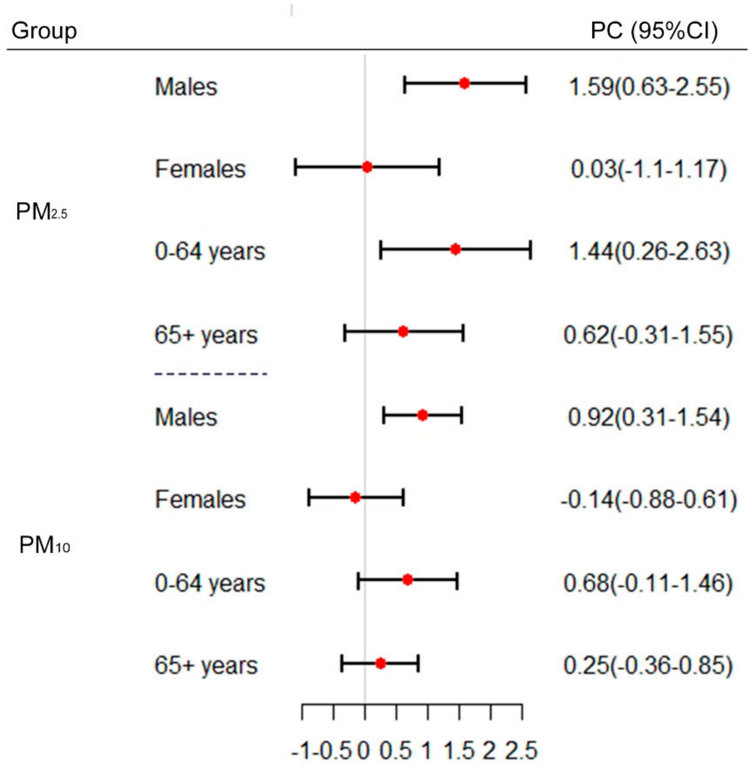

Figure S3. The estimated percent change of IHD hospitalizations per 10 μg/m^3 increase in $PM_{2.5}$ and PM_{10} concentrations, by gender and age group. PC: percent change; CI: confidence interval.

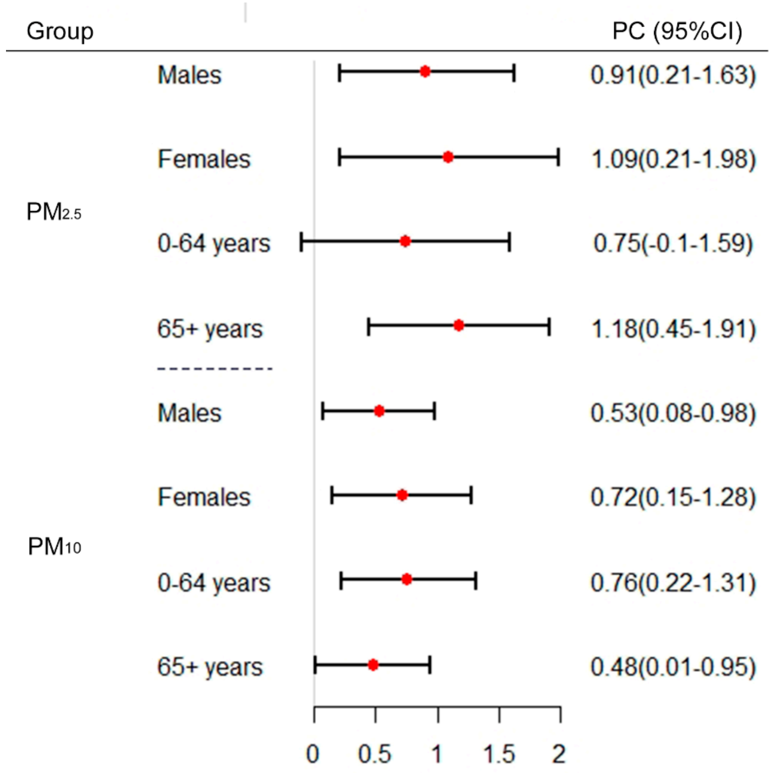

Figure S4. The estimated percent change of stroke hospitalizations per 10 μg/m^3 increase in $PM_{2.5}$ and PM_{10} concentrations, by gender and age group. PC: percent change, CI: confidence interval.

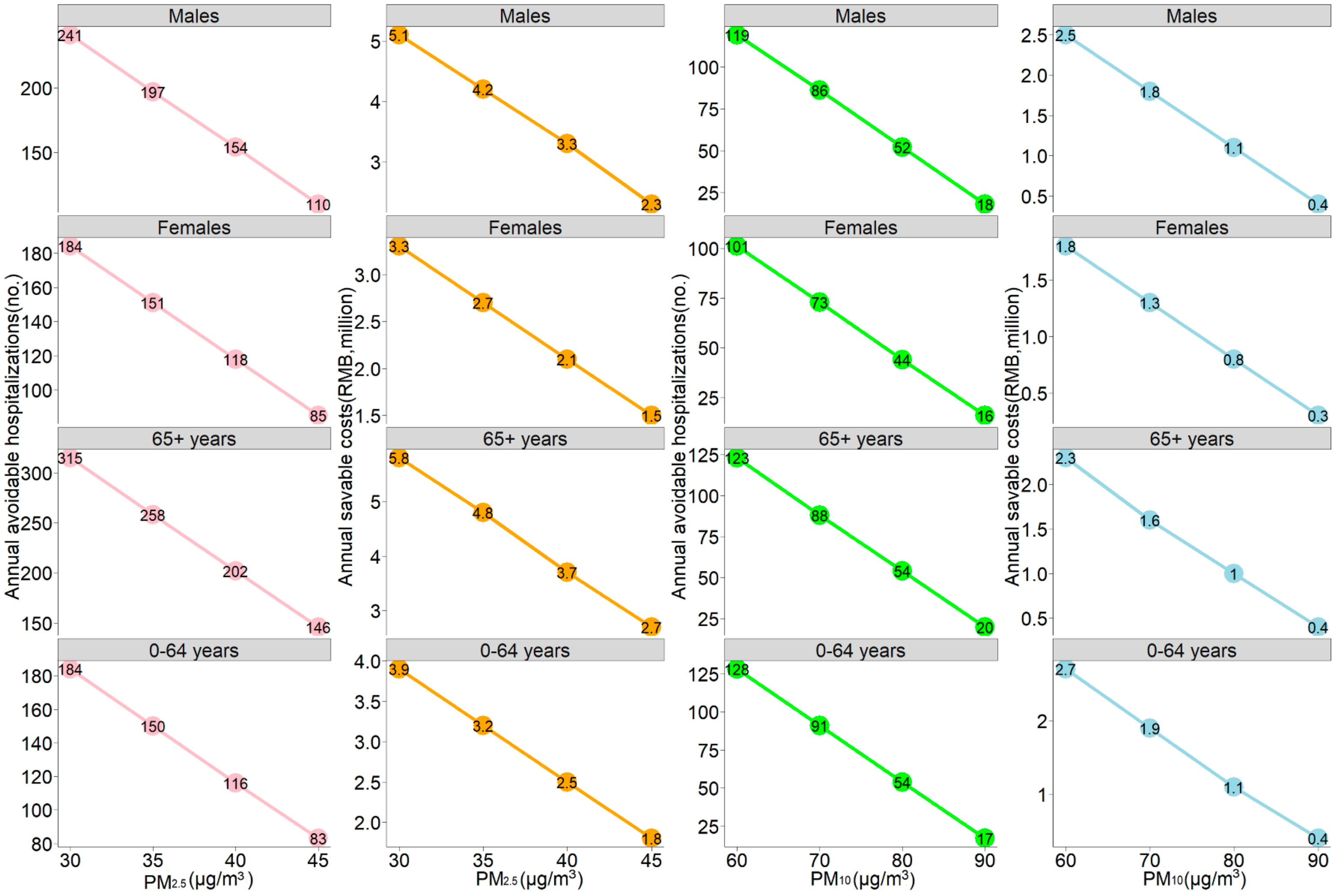

Figure S5. The estimated annual avoidable hospitalizations and savable hospitalization costs for CVD subgroups if the historical concentrations of $PM_{2.5}$ and PM_{10} could be maintained at relatively low levels. The air quality standard proposed by the WHO (24-h average value: 25 μg/m³ for $PM_{2.5}$ and 50 μg/m³ for PM_{10}) was considered as the reference.

Table 1. The coefficient of the Spearman rank correlation between particulate matter (including $PM_{2.5}$ and PM_{10}) and SO_2, NO_2 and meteorological factors in Wuhan, China.

Variable	SO_2	NO_2	Relative humidity	Mean temperature
$PM_{2.5}$	0.62 ***	0.62 ***	-0.53 ***	-0.07 *
PM_{10}	0.62 ***	0.68 ***	-0.32 ***	-0.28 ***

Notes: * $p < 0.05$, ** $p < 0.01$, *** $p < 0.001$.

Table 2. Results of sensitivity analyses by adjusting for co-pollutants and changing the degree of freedom for the long-term trend and seasonality. Results are shown in percent change (%) per 10 μg/m^3 increase in $PM_{2.5}$ and PM_{10} concentrations at the best lag day.

Variable	CVD		IHD		Stroke	
	$PM_{2.5}$	PM_{10}	$PM_{2.5}$	PM_{10}	$PM_{2.5}$	PM_{10}
Main model	1.01(0.67, 1.34)	0.48(0.26, 0.70)	1.10 (0.37, 1.84)	0.59(0.11, 1.07)	1.01 (0.45, 1.56)	0.61(0.26, 0.6)
Adjusting for co-pollutants						
$+SO_2$	1.10(0.74, 1.46)	0.58(0.33, 0.84)	1.04 (0.26, 1.83)	0.55(0.01, 1.11)	1.06 (0.46, 1.66)	0.69(0.29, 1.10)
$+NO_2$	1.39(1.01, 1.78)	0.83(0.56, 1.10)	1.18 (0.33, 2.20)	0.67(0.66, 1.27)	1.21 (0.56, 1.85)	0.82(0.38, 1.27)
Changing degree of freedom						
df=8	0.97(0.63, 1.31)	0.40(0.18, 0.62)	1.31 (0.40, 1.87)	0.56(0.08, 1.04)	0.99 (0.43, 1.55)	0.56(0.21, 0.92)
df=9	0.91(0.57, 1.26)	0.37(0.15, 0.60)	0.99 (0.24, 1.74)	0.48(0.01, 0.95)	0.94 (0.37, 1.15)	0.55(0.19, 0.91)
